# Single-trial classification of gait and point movement preparation from human EEG

**DOI:** 10.3389/fnins.2013.00084

**Published:** 2013-06-11

**Authors:** Priya D. Velu, Virginia R. de Sa

**Affiliations:** ^1^Department of Neurosciences, University of CaliforniaSan Diego, La Jolla, CA, USA; ^2^Department of Cognitive Science, University of CaliforniaSan Diego, La Jolla, CA, USA

**Keywords:** EEG, gait intent, point intent, brain-computer interface

## Abstract

Neuroimaging studies provide evidence of cortical involvement immediately before and during gait and during gait-related behaviors such as stepping in place or motor imagery of gait. Here we attempt to perform single-trial classification of gait intent from another movement plan (point intent) or from standing in place. Subjects walked naturally from a starting position to a designated ending position, pointed at a designated position from the starting position, or remained standing at the starting position. The 700 ms of recorded electroencephalography (EEG) before movement onset was used for single-trial classification of trials based on action type and direction (left walk, forward walk, right walk, left point, right point, and stand) as well as action type regardless of direction (stand, walk, point). Classification using regularized LDA was performed on a principal components analysis (PCA) reduced feature space composed of coefficients from levels 1 to 9 of a discrete wavelet decomposition using the Daubechies 4 wavelet. We achieved significant classification for all conditions, with errors as low as 17% when averaged across nine subjects. LDA and PCA highly weighted frequency ranges that included movement related potentials (MRPs), with smaller contributions from frequency ranges that included mu and beta idle motor rhythms. Additionally, error patterns suggested a spatial structure to the EEG signal. Future applications of the cortical gait intent signal may include an additional dimension of control for prosthetics, preemptive corrective feedback for gait disturbances, or human computer interfaces (HCI).

## Introduction

The detection of locomotive intent has potential as a control signal in brain-computer interfaces, for mitigating complications in movement disorders, or for use in environments with human computer interfaces (HCI). Detection of this signal is valuable for spinal cord injury patients and other users of lower limb prosthetics or artificial exoskeletons. It may also prove useful for alleviating abnormal states that prevent proper gait coordination such as during freezing of gait in Parkinson's disease by providing preemptive visual cues that help prevent freezing episodes (Hanakawa et al., 1999; Jiang and Norman, 2006; Nieuwboer, 2008). Other uses could be in HCI systems that anticipate user movement. Truly useful and versatile applications will require differentiation between many possible types of movements as well as the directionality of the movements. Here we explore the possibility of electroencephalography (EEG) to differentiate the intent to produce a locomotive movement from another movement as well as the spatial direction of the movement.

EEG is an ideal modality to capture locomotive intent. Its non-invasive nature is attractive to users, and the high temporal resolution, which is lacking in other non-invasive methods such as functional magnetic resonance imaging (fMRI), is suitable for capturing the cortical dynamics of gait planning and production. EEG systems are also inexpensive compared to other imaging technologies and can be worn comfortably by the subject even during large-scale movements such as locomotion. There are many groups competing to produce commercial dry electrode EEG systems, including ones that wirelessly connect to cell phones (Matthews et al., 2008; Yasui, 2009; Wang et al., 2011).

Locomotive movement through an environment is a crucial survival trait of many organisms. Vertebrates perform locomotive movements through a multi-level control system involving the cortex, brainstem, cerebellum, and spinal cord. This system integrates visual, vestibular, and proprioceptive information from the environment to perform coordinated movement of joints and muscles. Direct pathways from the cortex to the spinal cord have been shown to be vital for skilled movement, and pathways from the cortex to the basal ganglia may be important for online change of gait in response to obstacles or new goals (Graybiel, 2005; Grillner et al., 2008). Recently, intracortical recordings from hundreds of neurons in monkey motor cortex were successfully decoded to 3D coordinates of leg joints during treadmill walking of varied speed and direction (Fitzsimmons et al., 2009). A similar experiment conducted with humans and using EEG decoded the linear and angular kinematics of the ankle, knee and hip joints during treadmill walking (Presacco et al., 2011).

Neuroimaging studies in humans provide further evidence that the cortex is active during gait preparation and gait production. These studies attempted to isolate the neural correlates of bipedal gait production in humans by measuring brain activity during visual observation and mental motor imagery of gait and related motor activities with fMRI and positron emission tomography scanning (PET) (Malouin et al., 2003; Jahn et al., 2004; Bakker et al., 2008; Iseki et al., 2008; Wang et al., 2008, 2009). Groups have also recorded brain activity during gait movement with near infrared spectroscopy (NIRS) or EEG to determine brain regions involved in gait, to demonstrate effective movement artifact elimination from EEG, and to characterize the EEG signal during gait production (Yazawa et al., 1997; Miyai et al., 2001; Gwin et al., 2010, 2011; Wagner et al., 2012).

This study aims to detect EEG based cortical activity that is related to gait preparation before the voluntary motor production of gait. To our knowledge, we are the first to attempt to classify the intent to walk from single trial EEG data recorded before the onset of a natural gait movement in which the subject walks in real space from a starting position to a target position. We also classify the intent to walk from another motor plan, the intent to point. The classification of reach preparation before movement onset from EEG has been well established (Hammon et al., 2008; Wang and Makeig, 2009; Lew et al., 2012).

The classification process here uses information from all available frequency ranges and channels to allow for individual variations in cortical topography and dynamics. By examining which channels and which frequency ranges are most weighted by the top principal components and the LDA classifier, we characterize the nature of the signal used in classification. Finally, we attempt classification of pre-movement EEG between different target positions located in spatially distinct areas either to the left, right or in front of the subject.

We used a feature space that could capture mu (8–13 Hz) and beta (14–25 Hz) frequency band desynchronization over central motor and premotor areas (PMAs) as well as slow movement related potentials (MRPs) within single trials. Wavelets, especially the Debauchies (db) family, have been extensively used for EEG classification. The intended motor plan was predicted by classification of a principal components analysis (PCA) reduced feature space using regularized linear discriminant analysis (rLDA).

## Materials and methods

### Data collection

Nine healthy, right-handed subjects (18–27 years old, 2 females) participated in the experiment. All subjects read and signed informed consent forms that were approved by the UCSD Human Research Protections Office. Subjects were naive and untrained in the task and no feedback was given that could cue subjects to modulate their cortical signals to produce better features over time.

Continuous EEG was recorded from 64 Ag/AgCl electrodes positioned on a BioSemi nylon head cap according to the 10–20 International System. The signal was amplified with fixed gain BioSemi ActiveTwo amplifiers, band-passed from 0.2 to 100 Hz, and digitized at 512 Hz with 24-bit resolution. The independent software package DataRiver was used to read and record EEG signals as well as to integrate EEG signals with events from the Stim2007 stimulus presentation software (Delorme et al., 2011, 2012; Vankov et al., 2010). Two EOG electrodes were placed to record eye movements (one on the right outer canthus and one below the right eye). Right and left mastoid electrodes were averaged off-line to serve as reference. To minimize movement artifacts, subjects were encouraged to remain as still as possible and to look at a fixation cross on the wall until they heard the go cue. Two electrodes were placed on the anterior tibialis muscle (the first muscle to activate in gait) on each leg to detect premature muscle contraction during trials (Mann et al., 1979). As only eight EMG electrodes were available, the muscle activity of the arm could not be monitored through electrophysiological methods; the experimenter noted and discarded any trials with premature pointing or arm movements unrelated to the task.

**Figure 1:**
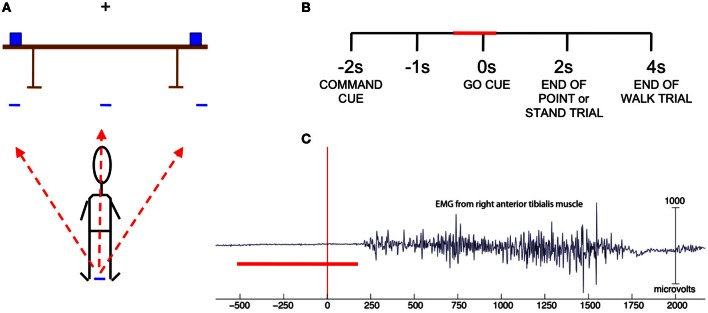
**(A)** Experiment set-up. Subject stands with arms at side and fixates on cross bar until the go cue sounds to perform one of six actions: walk left, front or right to target marked on floor, point left or right at an object on the table, or stand still. **(B)** Trial structure with the red bar indicating time range of data used in classification. **(C)** EMG from right anterior tibialis muscle of one subject. Trials in which EMG indicated movement onset prior to 200 ms were omitted. The red bar indicates the time range of data used in classification.

Cues were given in the form of auditory stimuli played through two speakers located behind the subject. A trial consisted of a command cue spoken by a computer-generated voice (walk front, walk right, walk left, point right, point left, stand still) followed by a delayed go cue (indicated by an auditory tone of 1 s in duration). The command cues were 1 s in length, and an interval of 1 s occurred after the end of the command cue and before the sound of the go cue. For point and stand trials the end of the trial was indicated by an auditory tone 2 s after the go cue while for walk trials the same tone was sounded 4 s after the go cue (Figure 1B). The experiment consisted of 60 trials for each condition, or a total of 360 trials, presented in pseudorandom order in six blocks of 60 trials with 2 min of rest between blocks, with the exception of Subject 1, who had 80 trials per condition. We reduced the number to 60 trials per condition for subjects 2–9 as the time to complete the experiment was prohibitively long. After the go cue sounded, the subject either walked forward five feet to designated spots on the floor to the right, left, or in front of standing position; pointed at designated objects on the right and left ends of a table placed five feet in front of the subject, or remained standing still with eyes focused on the fixation cross at eye level on the wall directly in front of the subject (Figure 1A). The subject carried the EEG amplifier and battery in a specially designed backpack for the duration of the experiment.

### Data analysis

All data were analyzed offline. For artifact removal, data were high pass filtered above 1 Hz to remove slow cortical potentials and galvanic skin potentials. The experimenter first visually inspected the data for removal of noisy channels, epochs with artifacts, and epochs with incorrect responses or premature leg movement (Figure 1C). These data was then further cleaned using EEGLAB automatic artifact rejection functions that removed channels and epochs that had kurtosis measures 5 standard deviations from the mean kurtosis value (Delorme and Makeig, 2004; Delorme et al., 2011). Kurtosis is the fourth moment measure of a probability distribution, and large positive kurtosis values indicate increased peaky shape whereas large negative kurtosis values indicate abnormally flat shape in the distribution. In EEG, these may represent undesirable artifacts in the data.

The above channels and time points were noted and then excluded from original raw, unfiltered data for subsequent classification analyses. The number of trials remaining for each class and the number of channels remaining for each subject after artifact rejection are shown in Table 1.

**Table 1:** **The number of channels and the number of trials for each class remaining after artifact elimination are shown for each subject**.

**Subject**	**Channels**	**L walk**	**R walk**	**F walk**	**L point**	**R point**	**Stand**	**Cut-off (%)**
S1	63	66	66	66	66	66	72	41
S2	59	44	44	46	44	44	46	40
S3	59	49	49	49	49	49	50	40
S4	51	45	45	52	45	45	49	40
S5	62	48	48	48	48	48	58	40
S6	58	36	36	36	36	36	51	39
S7	50	27	27	27	27	27	29	37
S8	50	44	44	44	44	44	48	40
S9	59	47	47	47	47	47	47	40

The final column lists the adjusted cut-off for binary classification significance based on Wald intervals modified for small sample sizes determined by the class with the lowest number of trials.

Filtering of data provided to the classifier was explicitly avoided so as to avoid any misrepresentation or distortion of the signal. High pass and acausal filters are commonly used in EEG research to remove noise, but these are poor choices for predictive classification as they produce temporal smearing of the signal such that values at future time points in which actual movement occurs may be used in the calculation of values at time points of interest for movement prediction. Causal filters avoid this problem, but still cause distortions in the original signal.

For classification, EEG data were separated into epochs starting 500 ms before and ending 200 ms after the onset of the go cue (but before the onset of gait) of each trial. We used this time range in order to capture the MRP signal from the motor cortex as well as any pre-movement related mu or beta desynchronization. Epochs were then processed to extract wavelet coefficients as the features for the classifier.

### Rationale behind feature selection

Wavelets provide high-resolution frequency information at low frequencies and high-resolution time information at higher frequencies. Many biological signals consist of slow oscillating background activity with rapid onset of change in activity. The temporal dynamics of such frequency perturbations may be better captured by wavelets than other methods.

Wavelet decomposition was performed on all available channels within a subject. This yielded a high dimensional feature space with *d* = *n* channels × 358 coefficients per trial. To facilitate classification by LDA, this high dimensionality was reduced by PCA to ten dimensions per trial (Lan et al., 2010). We used all 10 principal components for classification since only components with the highest variance were not ensured to be informative for classification (Lugger et al., 1998). Thus, we let the classifier decide which components were most informative by assigning those components higher weights during the training process.

### Feature parameters

We calculated the coefficients from level 9 discrete wavelet decompositions of the data using Daubechies wavelet 4 (db4) as the mother wavelet with periodization padding. The db family of wavelets has been shown to be better than biorthogonal wavelets, autoregressive filtering, and mu-matched filtering for extracting movement-related information from EEG signals (Renfrew et al., 2008). The db4 wavelet has been used extensively to analyze EEG signals (Subasi, 2006) and has performed as well as wavelets customized to individual subjects' EEG training data (do Nascimento and Farina, 2006; Farina et al., 2007) in a task predicting torque direction in foot movement.

Since the EEG signal was sampled at 512 Hz, the Nyquist frequency was 256 Hz. The level 1 decomposition thus included frequency information from 128 to 256 Hz, the level 2 decomposition from 64 to 128 Hz, and so on (Table 2). Detail coefficients from levels 1 to 9 encompassed available frequency signals from 0.5 to 56 Hz, and the approximate coefficients for level 9 had signals from 0 to 0.5 Hz. This range included both the mu and beta frequency sub-bands associated with idle motor rhythms and slow cortical potentials such as the MRP.

**Table 2:** **Wavelet levels and coefficients with corresponding frequency ranges**.

**Level**	**Frequency (Hz)**	**Coefficients**
9	0–0.5	1 (approximate)
9	0.5–1	2 (detail)
8	1–2	3, 4
7	2–4	5–7
6	4–8	8–13
5	8–16	14–25
4	16–32	26–48
3	32–64	49–93
2	64–128	94–183
1	128–256	184–362

### LDA classification

Features were extracted for six classes of trials: walk left, walk right, walk front, point left, point right, and stand. The classes were paired into 15 different binary classification problems, which can be separated into four different categories: same action with different directions, different actions with same direction, different actions with different directions, and actions vs. standing. Classes were also collapsed over directions to test the ability to classify walk/stand, point/stand, and walk/point.

A regularized LDA classifier (with optimized regularization parameter k) was trained on 10 features per trial using a 10-fold cross validation scheme (Friedman, 1989). Each class was assigned an equal number of trials and these trials were then randomly shuffled and partitioned into 10 sets, with nine used for training and one used for testing. Training and testing was done 10 times with a different set used each time as the test set. Thus, test data were completely separate from the training data. This complete process was repeated 9 additional times, and the prediction errors from the resulting 100 test cross validation folds were averaged and reported as the final results.

In situations with few trials compared to features, we do not have enough data to accurately estimate the covariance matrices used by LDA. In the absence of adequate information, a spherical covariance matrix is often assumed. Regularization provides a way to smoothly interpolate (using regularization constant *k*) between the sample covariance matrix and spherical covariance matrix. Within each fold, the optimal value for the regularization constant *k* was chosen from 10 values covering the interval [0.09 0.5] based on the value with the highest performance in an inner 4-fold cross validation scheme that used training data only.

Classifier performance was evaluated based on the number of trials used in classification. Chance level in a binary classification problem is not exactly 50%, but 50% with a confidence interval for a given *p*-value depending on the number of trials. The Wald interval is a normal approximation of the binomial confidence interval. As we had a small number of trials, we calculated Wald intervals with adjustments for a small sample size by adding four dummy observations, or two for each type (Agresti and Caffo, 2000; Müller-Putz et al., 2008). These intervals were then used to determine if the classifier performed significantly above chance or not.

### Location and frequency source analysis using PCA results

To better understand the nature of the signal used in classification and to ensure that classification was not based on eye or muscle movements, the final weights (all 10 PC weights multiplied by their respective LDA weights) were visualized with respect to space (channel scalp topography) and frequency band (wavelet coefficients.) The topographies of the final weights were plotted at each frequency band as well as averaged over all frequency bands to pinpoint the channels that contributed most to classification. The absolute values of the final weights were plotted for each wavelet coefficient of each channel to visualize which region of the frequency spectrum was most informative for successful classification. Scalp topographies from each of the 10 PC components averaged over all 100-folds and all frequencies from the subject with the best performance were plotted in order of the PC with the highest contribution to LDA to the PC with the lowest to determine if PCs with higher eigenvalues contained the most pertinent information for classification.

### Contribution from EOG and peripheral scalp channels

As an additional measure to assess the contribution of eye and muscle movements to the classification, LDA classification was performed using only EOG channels and those channels that would most be influenced by eye movements or muscle movements: Fp1, AF7, F7, FT7, T7, TP7, P9, P7, PO7, O1, Iz, Oz, O2, PO8, P8, P10, TP8, T8, FT8, F8, AF8, Fp2, and Fpz. These channels were located on the periphery of the EEG cap and were close to the origin of eye movements and muscles, and thus probably most strongly reflected the activity from these artifacts when compared to the other scalp channels.

Subjects with greater than chance performance based on the average of 10 runs of 10-fold CV from a specified LDA classification using this subset of channels were eliminated from the reported grand averages over subjects for that classification.

### Error patterns

To determine if there was underlying spatial structure in the EEG signal, multiple discriminant analysis (MDA) using the one class vs. rest scheme was applied to the following classification problems: (1) left walk, front walk, right walk (three classes); (2) left point, front walk, right point (three classes); and (3) left walk, left point, right walk, right point (four classes). To visualize classification performance, trials from all cross validation folds were grouped based on true class and predicted class. For example, in a three-class problem, the groups were arranged into a 3 × 3 confusion matrix with row labels corresponding to true classes and column labels corresponding to predicted classes. Cells were normalized by dividing by the total number of trials in the row class, so that the value of one cell was the fraction of trials that the classifier predicted to be in the class as defined by the column location of the cell.

## Results

### LDA classification

Individual subject errors and errors averaged over all nine subjects are shown in Tables 3A–3D for the 15 classification problems that consider direction as well as action. Mean and individual subject errors are shown in Table 4 for the three classification problems that collapse trials across directions and only consider differences in actions. Classification was successful if it yielded an error lower than the calculated threshold that was based on the number of trials used (Table 1).

**Table 3A:** **Classification errors for different direction, same action**.

**Subject**	**L walk/R walk**	**L walk/F walk**	**F walk/R walk**	**L point/R point**
S1	0.07	0.09	0.14	0.15
S2	0.37	0.42	0.41	0.36
S3	0.12	0.08	0.23	0.20
S4	0.15	0.01	0.23	0.27
S5	0.06	0.03	0.20	0.12
S6	0.32	0.37 (0.36)	0.46	0.41
S7	0.31	0.47	0.48	0.51
S8	0.07	0.08	0.14	0.11
S9	0.31	0.31	0.39	0.32 (0.35)
Mean	0.20	0.19	0.30	0.27
*p*-value	0.0005	0.0010	0.0011	0.0014

Above chance performances in classification using only EOG +peripheral scalp channels are shown in parenthesis next to the reported performances using only scalp channels. These values are not included in the calculation of the mean, and are crossed out.

**Table 3B:** **Classification errors for same direction, different action**.

**Subject**	**L walk/L point**	**R walk/R point**
S1	0.30	0.31
S2	0.41	0.43
S3	0.38	0.42
S4	0.24	0.26
S5	0.44	0.53
S6	0.41	0.39
S7	0.44	0.40
S8	0.39	0.36
S9	0.30	0.30
Mean	0.37	0.38
*p*-value	0.0003	0.0010

There were no above chance performances in classifications using only EOG +peripheral scalp channels.

**Table 3C:** **Classification errors for different direction, different action**.

**Subject**	**L walk/R point**	**R walk/L point**	**F walk/R point**	**F walk/L point**
S1	0.05	0.12	0.10	0.09
S2	0.39	0.35	0.39	0.33
S3	0.10	0.05	0.10	0.13
S4	0.03	0.05	0.07	0.06
S5	0.13	0.04	0.09	0.06
S6	0.25	0.30	0.36	0.43
S7	0.45	0.34	0.41	0.47
S8	0.11	0.07	0.07	0.12
S9	0.24	0.32	0.29	0.34
Mean	0.20	0.17	0.20	0.23
*p*-value	0.0001	0.0001	0.0002	0.0016

There were no above chance performances in classifications using only EOG +peripheral scalp channels.

**Table 3D:** **Classification errors for action vs. standing**.

**Subject**	**L walk/stand**	**R walk/stand**	**F walk/stand**	**L point/stand**	**R point/stand**
S1	0.12	0.16	0.20	0.16	0.25
S2	0.38	0.33	0.42	0.46	0.48
S3	0.25	0.24	0.20	0.13	0.15
S4	0.19	0.36	0.33	0.10	0.07
S5	0.16	0.27	0.05	0.08	0.05
S6	0.27	0.49	0.37	0.36	0.35 (0.32)
S7	0.47	0.47	0.41	0.39	0.55
S8	0.10	0.32	0.17	0.10	0.16
S9	0.24	0.35	0.27	0.24	0.28
Mean	0.24	0.35	0.27	0.25	0.28
*p*-value	0.0001	0.0007	0.0003	0.0002	0.0030

Above chance performances in classification using only EOG +peripheral scalp channels are shown in parenthesis next to the reported performances using only scalp channels. These values are not included in the calculation of the mean, and are crossed out.

**Table 4:** **Classification errors for combined directions**.

**Subject**	**Walk/stand**	**Point/stand**	**Walk/point**
S1	0.10 (0.38)	0.30 (0.39)	0.24
S2	0.34 (0.38)	0.41	0.40
S3	0.21	0.25	0.39
S4	0.19 (0.33)	0.22 (0.36)	0.24
S5	0.21	0.26	0.40
S6	0.35	0.41	0.40
S7	0.22 (0.34)	0.28	0.42
S8	0.18	0.17	0.28
S9	0.24	0.32	0.28
Mean	0.24	0.30	0.34
*p*-value	0.0005	0.0005	0.0002

Above chance performance in classifications using only EOG +peripheral scalp channels are shown in parenthesis next to the performance using all scalp channels. These values are crossed out, and not included in the calculation of the mean.

Only 9/162 classifications had significant performance using EOG and peripheral channels: L walk/F walk and R point/stand in subject 6; L point/R point in subject 9; walk/stand in subjects 1, 2, 4, 7; and point/stand in subjects 1 and 4. Within these nine classifications, the performance of EOG and peripheral channels was either poorer (8/9) or similar (1/9) to that of all scalp channels.

Left tailed one-sample *t*-tests using subject errors as samples, α = 0.05 and a mean set of 50% resulted in *p*-values below 0.05 for all 18 binary classifications. One-Way ANOVA testing suggested significant differences in means between the 15 classification problems that accounted for both direction and action (*p* = 0.035) and between the three classification problems that accounted for action only (*p* = 0.019). *Post-hoc* multiple comparisons test using Tukey's honestly different significance criterion both with and without assuming that the data followed a normal distribution revealed that only the mean error for walk vs. stand was significantly lower than the mean error for walk vs. point.

### Contributions from channels and frequency ranges

To pinpoint the origin and nature of the signal that the classifier was using, the contributions of different frequency ranges and channels were visualized by plotting the weightings by different PCs and the LDA classifier. The results for subject 4 (S4) and subject 8 (S8) in the two most difficult classifications, L walk/L point and R walk/R point, and in the three classifications collapsed across directions best provided insight into the classification process. Both subjects had significantly low errors for all classifications, but S4 probably had contributions from EOG or peripheral channels in the walk/stand and point/stand classifications while S8 had no such contributions. S8 had much higher error in the L walk/L point and R walk/R point classifications compared to S4.

**Figure 2:**
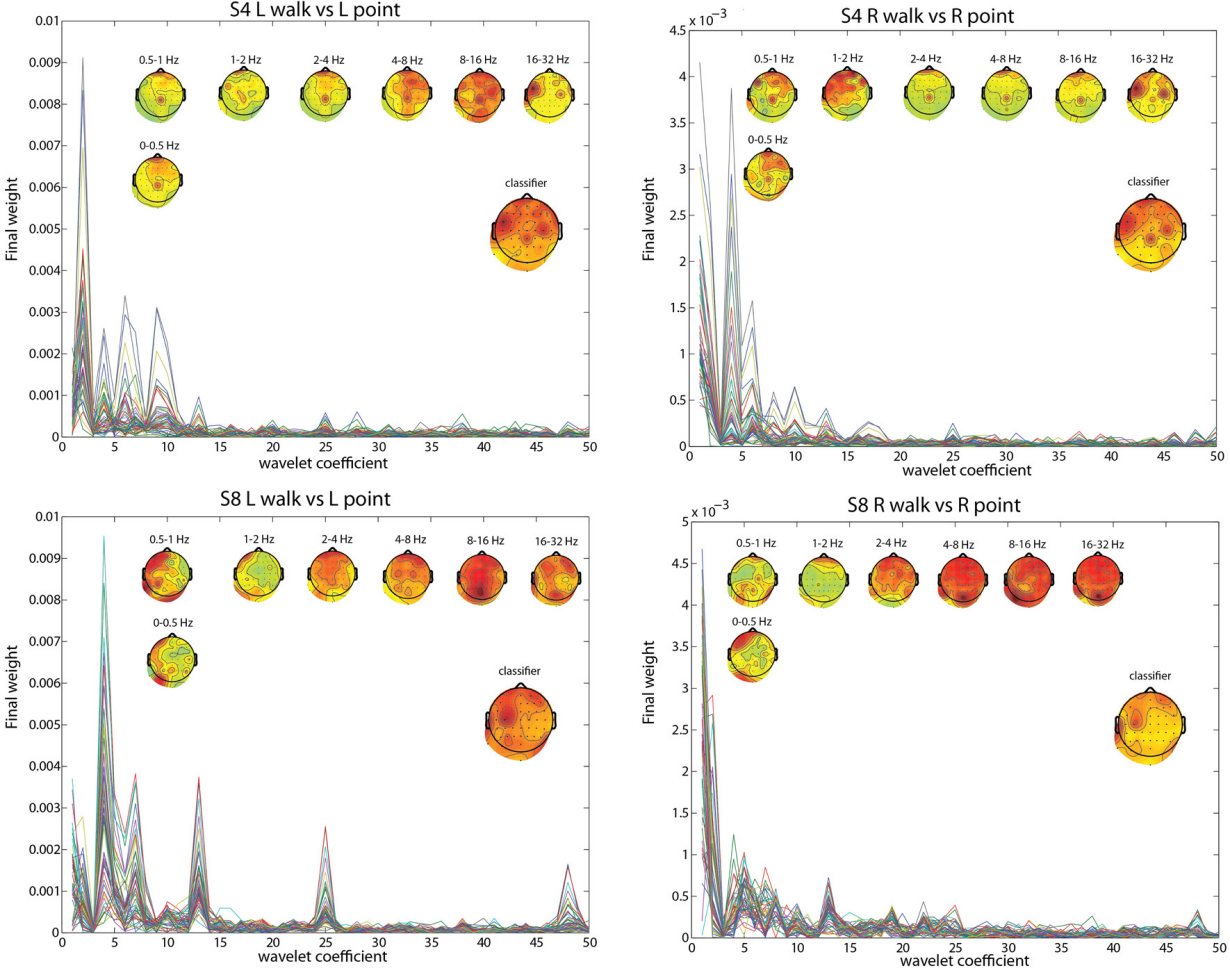
**L walk/ L point feature visualization for S4 and S8.** The smaller scalp maps show topographies of final weights of channels within different frequency bands. The larger scalp map shows the weighted average of the final weights from all frequencies, and represents what is used by the classifier. Each line represents the absolute value of the final weights for all coefficients for an individual channel.

**Figure 3:**
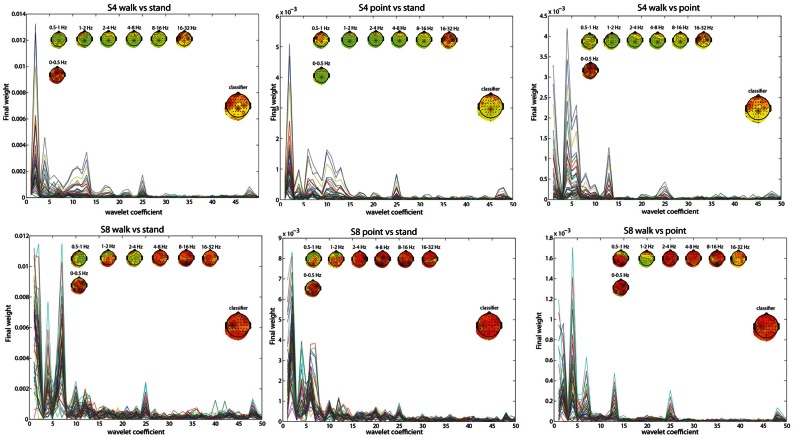
**Similar to Figure 2, but with walk/stand, point/stand, and walk/point feature visualization for S4 and S8**.

The frequency bands with the highest weighting were visualized by plotting the final weight for each of the 358 coefficients (Figures 2, 3). The final weight was computed by multiplying the PC weights from all 10 PCs by their respective LDA weights. This was done using training data from all 10-folds and 10 runs. Additionally, scalp topographies of channel weights were plotted using only coefficients within a given frequency band (smaller scalp plots) and using all coefficients (large scalp plot). The former indicated which brain regions had informative activity within a given frequency band while the latter gave a sense of which channels were most crucial for the LDA classification. The plots show that coefficients corresponding to lower frequency bands were more highly weighted. The scalp map averaged over all coefficients indicated that channels Cpz, C4, and FC5 were most highly weighted in S4 whereas S8 only had one channel, C3, highly weighted. The scalp maps within different frequency bands showed very variable topographies.

Using EOG and peripheral channels alone resulted in significantly low errors in the walk/stand and point/stand classifications in S4. Scalp topographies suggested weighting of the frontal electrodes in a pattern that suggested vertical eye movements, though this reliance on frontal electrodes was diminished in the walk/point classification. An example of possible neck muscle artifact can be seen in the scalp map for the 16–32 Hz frequency band of the R walk/R point classification. There were no scalp topographies with exclusively frontal or peripheral electrode weighting, which would suggest that eye or muscle movements were driving the classification.

**Figure 4:**
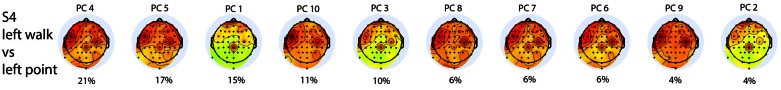
**Scalp topographies of the weighted average of the contributions from all frequencies for all 10 PCs used in the L walk/L point classification in S4 are ordered from the PC with the highest LDA weight to that with the lowest LDA weight.** Percentage contribution to LDA was calculated by normalizing LDA weights to one. PC1 has the highest eigenvalue and PC10 the lowest.

The most weighted PCs did not necessarily have the highest eigenvalues (Figure 4). There were three distinct topographies with slight variations represented by the 10 PCs, and the first five PCs summed to 75% of the total contribution to classification.

### Error patterns from MDA classification

**Figure 5:**
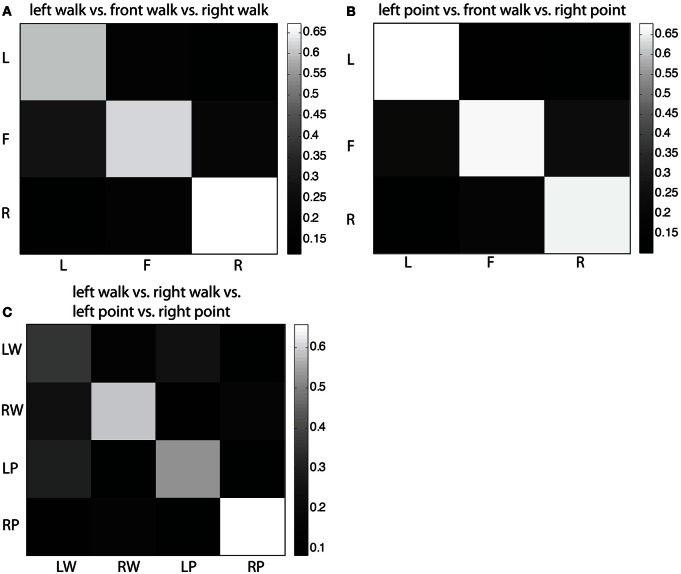
**(A)** Confusion matrix for three-class MDA of left walk/front walk/right walk. Row labels indicate true classes and column labels indicate classifier labels. Color of cells reflect fraction of trials classified as trial type indicated by the column label, i.e., the second cell in the top row indicates the fraction of left trials classified as front trials. Larger fractions (lighter colors) represent higher number of correct classifications. **(B)** Confusion matrix for three-class MDA of left point/front walk/right point. **(C)** Confusion matrix for four-class MDA of left walk/right walk/left point/right point.

In the confusion matrix for the three-class problem of left walk/front walk/right walk (Figure 5A), most left and right trials were correctly classified, less likely to be classified one location away, and least likely to be classified two locations away. Most front trials were correctly classified, with similar misclassification rates as left or right. This same structure more or less exists in the confusion matrix for the three-class problem of left point/front walk/right point (Figure 5B). In the four-class problem of left walk/right walk/left point/right point (Figure 5C), once again trials were most likely to be correctly classified. Direction was a stronger factor than type of action for correct classification. For example, left walk trials were more likely to be misclassified as left point trials but less likely and at similar rates to be misclassified as right walk or right point trials.

## Discussion

This study demonstrated that single trial EEG data is (1) classifiable for walk intent before the onset of natural movement, (2) classifiable between two motor plans (walking and pointing) that activate overlapping muscles, and (3) classifiable for an action at different target spatial locations. The largest contributors to successful classification were low frequencies (0–4 Hz) and channels located over areas involved in motor planning or motor production.

It is important to note that “movement intent” as used in this paper refers to the preparation of a movement by the subject in response to an external experimental cue. Further, movement was elicited by a warning stimulus (S1) that was followed by an imperative stimulus (S2). The resultant MRP from this paradigm is the CNV, which contains contributions from both motor preparation and attention for the upcoming stimulus (Luck and Kappenman, 2011). The motor aspect of the CNV is often equated to the late phase of the BP (Bereitschaftpotential), which is another slow cortical DC potential that is an indicator of human voluntary activity from as early as 2 s (the “early BP”) to 400 ms (the “late BP”) before the onset of movement (Shibasaki and Hallet, 2006). While the CNV results from external regularly timed cued movement, the BP results from internally self-paced movement. The beginning of the early BP starts in the pre-supplementary motor area (pre-SMA) and lacks somatotopic organization, but the rest of the early BP and the entire late BP have generators with discrete spatial locations corresponding to body parts. Application of this classifier paradigm to subject driven gait intent may show better classification as a result of the greater specificity of the BP compared to the CNV (Jankelowtiz and Colebatch, 2002; Lew et al., 2012).

A large challenge of using MRPs is finding informative activity within single trials since the low signal to noise ratio of a single trial presents a considerable obstacle in feature detection. Though the majority of MRPs in EEG literature are presented as ERPs averaged over hundreds of trials in the time-voltage domain, efforts are being made to capture the MRP within a single trial. One group succeeded in single trial detection of the MRP using features from wavelet analysis for predicting foot torque movement while subjects were seated (Farina et al., 2007).

Another feature commonly used in BCIs is the event related desynchronization (ERD)/event related synchronization (ERS), or the decrease/increase in power of mu (8–13 Hz) or beta (14–25 Hz) rhythms at somatotopically distinct regions before and during motor planning, action or imagery (McFarland et al., 2000; Pineda et al., 2000). In select subjects, it is visually observable during single trials and has been used to detect hand, foot, or tongue motor imagery (Pfurtscheller et al., 2006; Bai et al., 2008) or predict wrist extension (Bai et al., 2010). One study concluded that ERD and ERS was a more specific signal compared to the MRP for detection of pre-movement or motor imagery in a left hand/right hand/foot/tongue task (Morash et al., 2008). It is important to note that this feature varies spectrally and topographically in individual subjects (McFarland et al., 2000).

In this study we used all frequency information available in the signal by including coefficients from the entire wavelet decomposition. Previously mentioned fMRI, PET, and NIRS studies on gait found task-relevant activity in the supplementary motor cortex (SMA), medial primary motor cortex, and medial sensorimotor cortices. Cortical folding is different between individuals, which results in highly variable topographic distributions of useful EEG signal within a group of subjects. We created unsupervised custom spatial filters for each subject by including all noise-free channels available from the 64-channel montage covering the scalp and then applying PCA to transform and reduce this large feature space to the 10 dimensions with the most variance in the signal.

The variable ranking of PCs by LDA weights was in line with a study of single trial EEG classification using PCA-reduced feature space and LDA for an imagined left-and-right hand movement task (Lugger et al., 1998). Though the first few PCA components had the greatest variance, they were poor in discriminating between left vs. right hand motor imagery. These components were thought to represent background cortical EEG activity that was unrelated to the task.

Visualizations of the features used by the classifier revealed that frequency components within the range of the MRP were most heavily weighted. The channels most weighted in S4 may have reflected activity from the PMd (FC5), leg motor area (CPz), and arm motor area (C4), which could explain the lower errors for the L walk/L point and R walk/ R point classifications compared with S8, who only had high weighting of channel C3. Notably, the classification error for walk/point was similar for both S4 and S8, possibly because more trials were available for training as the classification used trials from both left and right conditions.

The dorsal premotor cortex (PMd) is postulated to be activated in externally cued movement preparation vs. movement that is internally driven by the subject, and vice versa in the SMA. Evidence for this has been circumstantial in humans, though one group demonstrated double-dissociation of PMd and SMA activity on MRPs by repetitive transcranial magnetic stimulation (rTMS) in humans during a right digit task in externally cued vs. internally driven conditions (Lu et al., 2011). Most relevant to this EEG study is an experiment that demonstrated that EEG surface potential configurations had the same order and same strength but longer duration over the PMA compared to the SMA in externally cued vs. internally driven right digit movement (Thut et al., 2000).

BCIs also must minimize or eliminate contributions from non-cortical electrical activity, such as from EOG and EMG. These signals not only obscure the cortical signal but can misdirect classification as in a situation when a subject is looking at a target that is not located in the intended direction of gait. Topographies suggestive of eye and muscle movements did appear in some scalp maps but were not the only feature present in the maps or had smaller weights. Classification using EOG and peripheral scalp channels resulted in significantly low errors in only 6% of all classifications, and these error values were poorer or comparable to those from classifications using only scalp channels. The classifiers appear to have minimized the contribution of eye or muscle movement on the scalp channel cortical signals for correct classification of movement intent.

Another challenge was in differentiating between two different motor plans to ensure that discrimination was based on a signal unique to gait movement intent and not generalized to any movement intent. Further, this was not a straightforward classification of an upper limb activity from a lower limb activity. Gait requires not only the participation of lower limb muscles, but trunk and upper limb muscles as well. In EMG studies, the posterior deltoid muscle was consistently activated in all 35 subjects in a walking task (Barthelemy and Nielsen, 2010), and contributed to horizontal abduction, external rotation, and depression at the shoulder as well as flexion and supination at the elbow during arm reaching (Vandenberghe et al., 2012). Though regions of the cortex involved in planning movements of these muscles have specific somatopic distribution, they are contiguously located on the sensorimotor cortex (Bakker et al., 2007) and may not be easily separable by the poor spatial resolution of EEG. This was probably the biggest factor in the higher errors for the walk vs. point classification problems compared to point vs. stand and walk vs. stand classification problems, though it is notable that the errors were still significantly below chance. With more training trials and the use of a non-linear classifier such as kernel support vector machines, these errors may be further lowered.

Finally, we tested the ability to predict the direction of movement toward a target either to the left, in front, or right of the subject. For all three targets, subjects initiated walking with the same leg and same arm, so classification was not based on contralateral cortical activation of the limb used. Previously, reaching to three different (left, right, center) locations using the same arm revealed an underlying structure in the pre-movement EEG signal that corresponded with target space (Hammon et al., 2008). Trials were most likely to be correctly classified, less likely to be classified one target away, and least likely to be classified two targets away. We found a similar underlying structure that corresponded to the target space in our data.

We should note that in an online real-time BCI, the classifier would be trained on initial data and then be tested on future data. This means it must be robust to non-stationarity in the signals that can cause drifts in the features over time. In this experiment there were not enough trials to create a large enough training set for adequate training and a separate (later) test set with an adequate number of trials to reliably assess the classifier accuracy, so we were not able to test this aspect.

In conclusion, this study has shown that the EEG signal can be used for predictive classification between walk, point and stand actions as well between different target directions for these actions. Spatial and spectral contributions were from areas involved in motor planning or production and mostly from low frequency cortical activity, with smaller contributions from mu and beta frequency bands. It remains to be tested whether this signal can be detected in real-time non-stationary data.

### Conflict of interest statement

The authors declare that the research was conducted in the absence of any commercial or financial relationships that could be construed as a potential conflict of interest.
